# Continuous Semi-autonomous Prosthesis Control Using a Depth Sensor on the Hand

**DOI:** 10.3389/fnbot.2022.814973

**Published:** 2022-03-25

**Authors:** Miguel Nobre Castro, Strahinja Dosen

**Affiliations:** Neurorehabilitation Systems, Department of Health Science and Technology, Aalborg University, Aalborg, Denmark

**Keywords:** myoelectric hand prosthesis, semi-autonomous control, grasping, computer vision, point cloud processing, object segmentation

## Abstract

Modern myoelectric prostheses can perform multiple functions (e.g., several grasp types and wrist rotation) but their intuitive control by the user is still an open challenge. It has been recently demonstrated that semi-autonomous control can allow the subjects to operate complex prostheses effectively; however, this approach often requires placing sensors on the user. The present study proposes a system for semi-autonomous control of a myoelectric prosthesis that requires a single depth sensor placed on the dorsal side of the hand. The system automatically pre-shapes the hand (grasp type, size, and wrist rotation) and allows the user to grasp objects of different shapes, sizes and orientations, placed individually or within cluttered scenes. The system “reacts” to the side from which the object is approached, and enables the user to target not only the whole object but also an object part. Another unique aspect of the system is that it relies on online interaction between the user and the prosthesis; the system reacts continuously on the targets that are in its focus, while the user interprets the movement of the prosthesis to adjust aiming. Experimental assessment was conducted in ten able-bodied participants to evaluate the feasibility and the impact of training on prosthesis-user interaction. The subjects used the system to grasp a set of objects individually (Phase I) and in cluttered scenarios (Phase II), while the time to accomplish the task (TAT) was used as the performance metric. In both phases, the TAT improved significantly across blocks. Some targets (objects and/or their parts) were more challenging, requiring thus significantly more time to handle, but all objects and scenes were successfully accomplished by all subjects. The assessment therefore demonstrated that the system is indeed robust and effective, and that the subjects could successfully learn how to aim with the system after a brief training. This is an important step toward the development of a self-contained semi-autonomous system convenient for clinical applications.

## 1. Introduction

The control of robotic hand prostheses at a human-like level of dexterity remains unsolved despite the recent advancements in human-machine interfacing (HMI) (Farina et al., 2014, 2021; Geethanjali, 2016; Yang et al., 2019). To achieve such capabilities, both robotic hardware and control methods need to be mature enough. Whereas, in terms of hardware, robotic hand prostheses can already match fairly well the versatility (e.g., Laffranchi et al., 2020) and even the number of Degrees-of-Freedom (DoF) of a human hand, the HMI methods still struggle to offer reliable control solutions for dexterous systems (Asghari Oskoei and Hu, 2007; Fougner et al., 2012).

The conventional approaches to prosthesis control are based on intent detection from surface electromyography (sEMG). The direct control used in most commercial hand prostheses is characterized by a direct mapping between a specific sEMG channel and a pre-defined prosthesis DoF. To access different DoF, the user needs to generate switching commands, which makes this approach slow, difficult, and non-intuitive, especially in the case of more advanced prostheses with many functions. Classification-based control relies on pattern recognition (Atzori et al., 2016; Geng et al., 2016) to recognize a predefined set of gestures and, similarly to direct control, operates the prosthesis sequentially but eliminates the need for explicit switching. Regression-based approaches seem to be the most promising as they enable simultaneous control of multiple DoFs, allowing the prosthesis to move naturally (Hahne et al., 2018). However, the reliable control is limited to two-DoFs (Hahne et al., 2014). Both regression and classification rely on training data, which has to be collected *a priori*, and require subsequent recalibrations (Ortiz-Catalan et al., 2014; Parajuli et al., 2019). Furthermore, the performance of these control strategies is affected by the factors that change muscle activation patterns (i.e., controller inputs) such as sweat, arm position, electrode shift, and force levels (Farina et al., 2014; Parajuli et al., 2019). Lastly, in these methods, the cognitive burden is on the user's side as he/she is responsible for explicit control of all functions. Together, these factors might have a negative impact on the acceptance rates of these devices (Østlie et al., 2012), which have been low for nearly four decades despite the advancements in research (Biddiss and Chau, 2007; Salminger et al., 2020).

One approach to address these challenges is to use multi-modal control strategies that offer potential for semi-autonomous prosthesis control (Jiang et al., 2012). The main idea is to equip a prosthesis with additional sensors that provide information about the environment, thereby allowing the system to perform some tasks automatically, under the supervision of the user. This reduces the cognitive burden of control and allows operating complex systems with simple command interfaces.

For instance, imaging sensors that can retrieve RGB data (Došen et al., 2010; Ghazaei et al., 2017; Gardner et al., 2020; Zhong et al., 2020), depth data (Markovic et al., 2014, 2015; Ghazaei et al., 2019; Mouchoux et al., 2021), and a combination of both (Maymo et al., 2018; Shi et al., 2020) can be used for prosthesis control. These exteroceptive sensors enable the prosthesis controller to estimate the shape, dimension, and orientation of target objects and use this information to adjust prosthesis configuration automatically.

Došen et al. (2010) employed an RGB camera and a laser depth sensor placed on a prosthesis to estimate the size and shape of a target object and select prosthesis grasp type and size appropriate for grasping the object. Markovic et al. (2014) exploited augmented reality (AR) glasses with stereovision to model target objects by fitting geometric primitives and to provide visual feedback to the user. In the next study, Markovic et al. (2015) replaced stereovision with a depth sensor and included an inertial measurement unit to additionally estimate and adjust the prosthesis wrist orientation. In a recent work, Mouchoux et al. (2021) advanced the system further by enhancing depth data processing and using sensor fusion to enable the system to function in cluttered environments and without an explicit triggering of automatic operation.

Ghazaei et al. (2017) introduced the use of deep learning, namely convolutional neural networks, to classify images taken from an RGB camera and separate objects into categories corresponding to specific grasp types. The same authors (Ghazaei et al., 2019) later proposed to extend this classification approach using depth data, so that similar object shapes could be grouped in different grasp types based on the similarity of point cloud features. Zhong et al. (2020) used Bayesian neural networks to recognize target objects from RGB images, even in cluttered scenes, and quantify the uncertainty of the respective predictions. Shi et al. (2020) acquired an RGB-D dataset of objects for grasping tasks and found out that gray-scaled images plus depth data (both 2D-tensor inputs) improved the classification accuracy of grasp patterns compared to RGB data (3D-tensor), when using convolutional neural networks. Gardner et al. (2020) tested a framework for shared autonomy based on a multimodal sensor approach, where RGB data were used for object recognition while inertial data enhanced the intention prediction based on the subject's grasping trajectory.

Most of the aforementioned studies have presented systems where the vision sensors were placed on the user (Markovic et al., 2014, 2015; Gardner et al., 2020). This choice provides the best view in terms of scene-analysis but it requires the user to wear an additional component beyond the prosthesis [e.g., smart glasses with an embedded camera (Markovic et al., 2014) or an AR set (Mouchoux et al., 2021)]. A recent example presented in Mouchoux et al. (2021) describes a solution that provides extensive functionality (e.g., scene modeling, prosthesis tracking, and interaction prediction) but it is also rather cumbersome to wear. So far, only three systems have proposed a hand placement of the vision sensor (Došen et al., 2010; Ghazaei et al., 2017; Zhong et al., 2020), with a camera positioned on the dorsal side of a prosthesis or gripper. The advantage of such placement is that it resembles a self-contained system. However, it also represents a challenge as it limits the field-of-view of the camera and the sensor orientation depends on the prosthesis movements. In addition, the aforementioned self-contained approaches all relied on an RGB camera, which provides a limited functionality in terms of computer vision.

The semi-autonomous prosthesis control used in most of these studies relies on controllers that need to be explicitly triggered by the user. In such sequential scheme, a myoelectric command is performed to activate the controller to detect a given target object, process the visual information and adjust the configuration of the prosthesis to enable grasping. The semi-autonomous control has been shown to outperform direct control with switching (Markovic et al., 2015) as well as pattern classification (Mouchoux et al., 2021), especially in cases of complex systems with many DoFs. In addition, it also decreases the use of muscles during prosthesis control, thus reducing the physical effort.

The present manuscript proposes a novel, continuous semi-autonomous prosthesis control approach that uses a depth sensor placed on the dorsal side of the prosthetic hand. A closed-loop controller employs depth perception and enables the prosthesis to operate continuously by reacting to where the user aims at each moment. Thus, to grasp a target, the user needs to approach the object and point the camera. Once the target is detected and analyzed, the prosthesis automatically adjusts its configuration (wrist orientation, grasp type, and size) to align and pre-shape the hand for grasping. Importantly, the computer vision pipeline runs continuously, even while the prosthesis adjusts its configuration, and the controller is capable of perceiving not only whole objects but also their components (e.g., a tip) and segments/portions (e.g., a side). The automatic controller and the user operate the prosthesis in parallel, and the user interacts with the system by adjusting their aim to grasp a desired object part or an object lying within a cluttered scene. This is therefore, a closed-loop control scheme in which the user-system interaction (collaboration) is critical for successful task accomplishment. To investigate the development of these interactions in different scenarios as well as the feasibility of such a solution, the proposed system was tested in ten able-bodied individuals, who used the system to grasp individual objects or object parts placed on a table and within cluttered scenes.

## 2. Materials and Methods

### 2.1. System Components

A two-DoF left hand prosthesis (Michelangelo Hand, Ottobock, Duderstadt, Germany) was used in this study. The prosthesis is capable of rotating the wrist and opening/closing its fingers in two different grasp types—palmar (max. aperture 110 mm) and lateral (max. aperture 70 mm). The prosthesis was mounted on a forearm splint in order to enable testing in able-bodied participants.

The myoelectric interface comprised two double-differential surface EMG (sEMG) active/dry electrodes (Model 13E200 = 50, Ottobock, Duderstadt, Germany), which provided rectified and pre-amplified signals used for intent detection. The two sEMG channels were placed on the subject's right forearm (ipsilateral-side), above the wrist flexor and extensor muscles according to Cram's guidelines (Criswell, 2010). The communication between the prosthesis and the main processing unit (laptop) was established through a Bluetooth connection, which enabled reading of prosthesis sensor data (wrist angle, hand aperture, grasp type, and grasping force) and sEMG channels as well as sending velocity control commands to the prosthesis at a rate of 100 Hz.

The depth perception was provided by a Realsense™ active infrared stereo camera (Model D435i, Intel, US) using a depth stream with a resolution of 424 × 240 px and refresh rate of 90 fps. The camera was mounted on the dorsal side of the prosthetic hand and connected to a Microsoft Windows laptop (Intel^®^ Core™ i7-8665U CPU @1.90 GHz, 2.1 GHz, 4 Cores, 8 Logical Processors, with 32 GB RAM) using an extra 2 m active USB-A 3.0 extension cable (DELTACO Prime, SweDeltaco AB, Stockholm, Sweden).

### 2.2. Control Scheme

**Figure 1:**
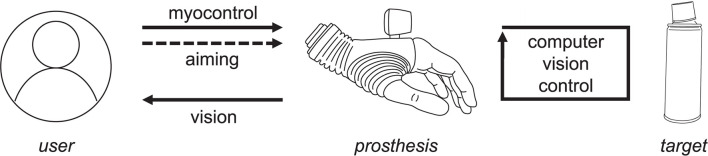
A semi-autonomous control strategy. The user orients the prosthesis to aim at the target. The prosthesis responds continuously to objects identified as the target, and the subject uses visual assessment and interpretation of the prosthesis motion to adjust the aiming.

The novel semi-autonomous control approach is depicted in Figure 1. The prosthesis is configured to start in autonomous mode, in which it reacts to the surrounding environment by continuously adjusting its configuration (wrist orientation, grasp type, and size), depending on the object or object part at which the user aims.

Computer vision closed-loop control uses depth sensing to continuously estimate the dimensions and orientation of a given target object or object part that is presently in the focus of the camera. This estimation is performed regardless of (1) the camera orientation (e.g., the hand being horizontal, tilted, or vertical), or (2) the side from which the target is approached. Based on the estimated object properties, the controller automatically orients and pre-shapes the prosthesis appropriately for grasping the identified target. Since the prosthesis continuously reacts to the environment, the user can rely on implicit visual feedback provided by the prosthesis movements to understand what the system is doing and adjust his/her aiming if required. Once the user is satisfied with the momentary prosthesis configuration, in which the system is aligned with the target and pre-shaped into a suitable grasp type and size, he/she can take over the control from the autonomous controller and proceed to grasping and manipulating the object using volitional direct control. The user takes over the control by extending the wrist, thus generating a myoelectric command. From that moment, the hand is controlled volitionally using direct proportional approach with two EMG electrodes, and the automatic control is reactivated by opening the hand and releasing the object.

**Figure 2:**
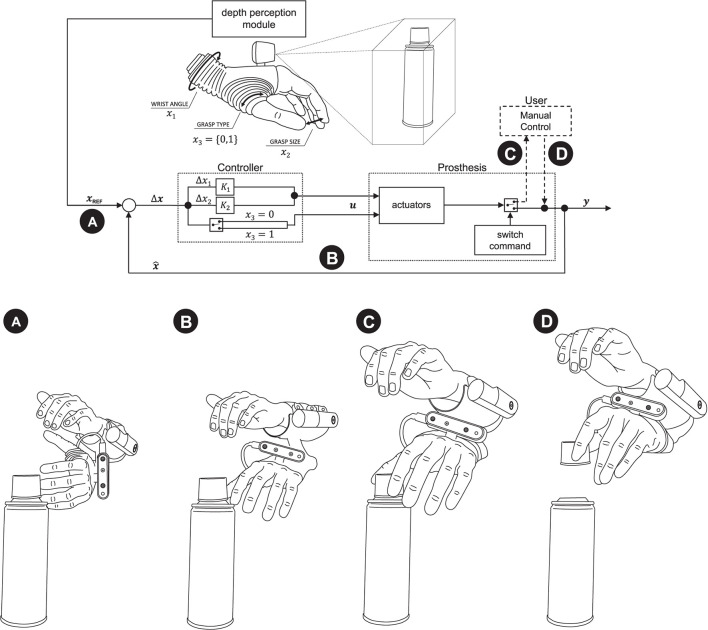
Semi-autonomous control scheme. A proportional controller **(B)** continuously adjusts the prosthesis configuration (***x***) to match the reference (***x_REF_***) provided by computer vision **(A)**. The computer vision module determines the reference configuration by estimating the properties of a target object. When the subject observes that the prosthesis is pre-shaped conveniently for the grasp, he/she takes over the control and closes the hand around an object **(C,D)**. Annotation: **x** and ***x_REF_***—the current and desired prosthesis state; ***u***—prosthesis commands; *x*_1_, *x*_2_, and *x*_3_—angle, grasp size, and type (0—palmar, 1—lateral).

The semi-autonomous control was implemented in C++ (Visual Studio 2019, Microsoft, US). The control loop is depicted in Figure 2 (top) together with an illustrative example (Figure 2, bottom). A proportional controller continuously changes the prosthesis configuration state ***x*** (until convergence) based on the momentary error (Δ***x***) with respect to the target configuration ***x_REF_***. The controller generates velocity commands (***u***) and the gains (*K*_1_ and *K*_2_) are adjusted heuristically to achieve a trade-off, namely, a responsive prosthesis that reacts to aimed objects but which, at the same time, does not move too fast so that the user can easily perceive and interpret the prosthesis behavior. The latter aspect is particularly important to close the control loop through visual feedback as indicated in Figure 1. The prosthesis state comprises three variables: two continuous (the wrist angle *x*_1_ and the grasp size *x*_2_) and one discrete (the grasp type *x*_3_). The wrist angle and grasp size are adjusted in normalized units, where [0, 1] interval corresponds to the range of motion, while the grasp type, takes two states, i.e., palmar or lateral grasp. The reference prosthesis configuration ***x_REF_*** is estimated from the depth data using computer vision as described in the next section.

In the example shown in Figure 2 (bottom panel), the hand is initially rotated so that the camera is vertical. The subject aims to the plastic cap at the top of a cylindrical can. The computer vision detects the object, rotates the hand using visual servoing (Figures 2A,B), selects the lateral grasp and adjusts the aperture size (hand horizontal, lateral grasp, small aperture). If at any moment during this process, the subject would aim a little below, the prosthesis would start configuring to grasp the can body (hand vertical, palmar grasp, medium aperture). The subject observes the hand motion, and when it assumes appropriate configuration, he/she takes over the control, closes the hand and lifts the object (Figures 2C,D).

### 2.3. Depth Perception and Object Modeling

**Figure 3:**
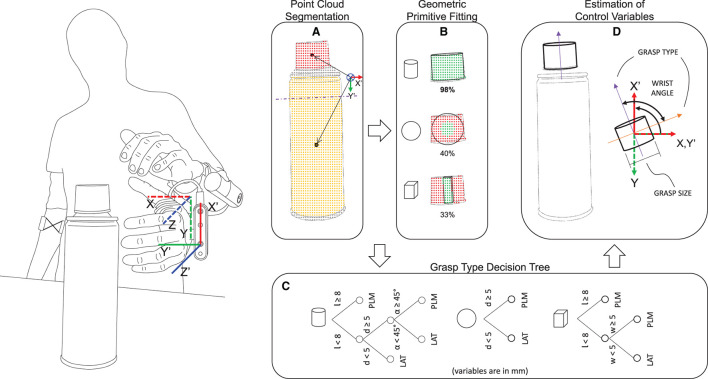
Depth perception pipeline. The scene is segmented in the point clouds belonging to different objects/object parts and the partitioned point cloud with the closest centroid **(A)** is selected for shape fitting **(B)**. In the present example, the plastic cap is chosen as the target and the best model to fit the point cloud is the cylinder. The coordinate system of the camera (X'Y'Z' frame, moving) and the socket (XYZ frame, static) is shown on the left. **(A)** Shows the projection of the point cloud centroids onto the X'OY' plane of the camera, and the arrows represent the distance of the centroids from the origin of the camera coordinate system. **(C)** The decision tree for the selection of grasp type uses object properties such as length *l*, diameter *d* (or width *w*) and approaching angle α (cylinder only), and enables selecting a palmar (PLM) or a lateral (LAT) grasp. **(D)** Derivation of the grasping direction vector from the model to calculate the orientation of the object in the static XYZ frame and the respective target wrist angle. The grasp size can be trivially obtained from the model.

The processing pipeline for depth data was implemented using the well-known open-source Point Cloud Library v11.0 (Rusu and Cousins, 2011). To decrease the amount of irrelevant depth data and prevent additional computational effort, the depth acquisition volume was confined to a virtual cropping box (hereafter referred to as “the scene”) with dimensions 150 × 150 × 250 mm. This volume was adopted by considering the task, namely, grasping objects in front of the hand. The data were downsampled using an approximate voxel grid with a voxel size of 2 × 2 × 2 mm. Given the hardware limitations imposed by the depth camera (minimum resolving depth distance) and the prosthesis fingers (violating that volume and appearing within the scene), the cropping box was positioned at a distance of 115 mm in front of the camera, i.e., along the z-axis of the hand/camera (Figure 3, left).

As shown in Figure 3 (left), the system considers two reference frames: one placed on the hand/camera (the moving frame X'Y'Z') and another placed at the prosthesis socket (the static frame XYZ). Since the socket reference frame is static in relation to the user's forearm, the system can identify a target object or object part regardless of the prosthesis or the user's arm orientation in the real-world. For example, the system will react exactly the same whether a target lies on a table, is placed on a wall or is hanging from the ceiling. This is an additional flexibility compared to the semi-autonomous systems presented in the literature (Markovic et al., 2014, 2015; Mouchoux et al., 2021), which often assume that the objects are placed on a supporting surface that is detected and then removed. In order to make the automated wrist motion more natural, the range of motion of the prosthetic wrist is limited to 70° of supination and 90° of pronation. Hence, when the prosthesis is fully pronated, the hand/camera X'Y'Z' frame aligns with the prosthesis socket XYZ frame.

The depth data processing pipeline (Figures 3A,B) involves three main steps: (1) point cloud segmentation, (2) target selection, and (3) geometric primitive fitting.

The segmentation is performed using the Locally Convex Connected Patches (LCCP) (Stein et al., 2014) algorithm that involves the clustering of distinct convex regions within the point cloud. The regions may correspond to isolated objects or different parts/portions within the same object (Figure 3A) and this enables the system to operate in cluttered environments. The segmentation output is a list of labeled centroids of the point clouds that fulfill a minimum size threshold.

The target selection step uses the list of labeled centroids and infers the aimed target by projecting all centroids onto the X'OY'-plane of the camera and selecting the one that is closest to the origin, as exemplified in Figure 3A. This grants robustness to the aiming process as the subject does not need to aim directly at an object, but to its vicinity.

Finally, the Random Sample Consensus (RANSAC) (Fischler and Bolles, 1981) algorithm is used to simultaneously (i.e., parallel processing with multiple threading) fit each of the three pre-defined geometric primitive models (a sphere, a cylinder, and a cuboid) to the selected point cloud and choose the best fitting model (Figure 3B).

While the RANSAC fitting of the parametric sphere and cylinder models is trivial, the cuboid primitive is obtained by fitting of up to two perpendicular plane models. These are consecutively fitted to the selected sub point cloud until more than 70% of the points in the point cloud are inliers. Once the point cloud of the first plane is obtained, the first two axes of the cuboid primitive are determined by applying the principal component analysis to the 3-D points to find the first two principal components. All points on that plane are then projected along these two axes to obtain the first two dimensions, width and height, of the cuboid primitive. If a second perpendicular plane can still be fitted to the remaining points of the sub-point cloud, the third axis is defined by the cross-product between the first two principal components. The third dimension of the cuboid primitive, the depth, is then found by projecting all points of the second plane's point cloud along that third axis. Otherwise, if only plane model could be fitted, the depth is considered to be 5 mm by default (“thin” object).

### 2.4. Estimation of Grasp Type, Size, and Wrist Orientation

The computer vision module estimates the desired prosthesis configuration (***x_REF_***) by considering the properties of the grasping target (object or object part) retrieved from the best fitting model. To decide the grasp type and size, the model properties (length *l* and diameter *d*, or width *w*) and approaching angle α (cylindrical model only) are processed by a decision tree (Figure 3C), one per model type, similarly to the approach initially proposed by Došen et al. (2010). The trees are constructed so that the long and/or thick objects are grasped using palmar grip, while lateral grip is used for smaller thin objects. In the case of cylindrical targets, an approaching angle α measured between the longitudinal axis of the cylinder and the Z'-axis of the prosthesis frame is used to understand if the cylinder is being approached frontally or from above, using an angular threshold of 45°. The approaching angle α allows the system to grasp a cap of a jar or a bottle, using a palmar grasp (as a surrogate of a three-finger pinch) when the cap is larger.

The cuboid model is however treated as a special case. This is not only to grant robustness to the system but also because this primitive can present itself with a single face or multiple faces. Therefore, in the presence of a full cuboid object with multiple faces, it is necessary to infer to which face the user aims at, as the grasping strategy depends on the dimensions (length *l* and width *w*) of the targeted face. For that reason, a ray casting procedure is implemented if two or three faces of the cuboid primitive can be seen from the aiming perspective. The approach comprises the following steps: (1) find the closest face among each pair of opposing faces of the cuboid and calculate its center; (2) find the intersection point between the Z-axis of the socket frame and each of the closest face planes; (3) calculate the distance vector from each face center to the respective co-planar intersection point; (4) verify if the intersection point is located outside the dimensions of the current face in the direction of an adjacent and perpendicular face; (5) if it is, reject the current face, otherwise select the face corresponding to the shortest distance vector. Once the face is selected, the longest dimension is set as length *l* and the smallest as the width *w*. The obtained parameters then allow selecting the grasp type using a dedicated decision tree.

Other control variables, namely wrist orientation and grasp size, are computed after the grasp type *x*_3_ has been selected. As illustrated in Figure 3D (purple arrow), the grasping direction vector is obtained from the local reference frame of the target's primitive model. This vector is by default set along the direction about which the length *l* of the model is measured. Thus, it corresponds to the longer axis of a cuboid model or to the longitudinal axis of a cylinder (as shown in the figure). In the case of a spherical model, the grasping direction vector assumes the direction of the X-axis of the prosthesis socket reference frame. As the palmar and lateral grasps of the Michelangelo hand differ by the positioning of the prosthesis thumb, the grasping direction vector is rotated clockwise (Figure 3D, orange arrow) in case lateral grasp is selected for the given target. The projection of the grasping direction vector onto the X'OY'-plane of the moving frame of the hand/camera defines a reference prosthesis wrist orientation angle *x*_1_ as measured from the socket reference frame (XYZ). Since the zero angle of the hand moving frame matches the full/maximum pronation (~90°), an horizontally oriented target that requires a larger pronation angle (with respect to the socket reference frame) will be interpreted as a supination angle, i.e., the grasping direction vector is automatically flipped 180°. This may therefore require a slight socket inclination, for instance, while picking a long object lying on a table.

The grasp size *x*_2_ is given by diameter *d* (spherical or cylindrical model) or width *w* (cuboid model) to which an offset is added to introduce a safe zone (aperture somewhat larger than the object size). A smooth transition between different grasps and aperture sizes, which can be triggered as the user reorients the prosthesis and/or camera moves, is ensured by applying a moving average filter with a window size of 20 samples, for the control variables *x*_1_ and *x*_2_, and majority voting window of 10 samples for the grasp type *x*_3_.

### 2.5. Participants

Ten able-bodied participants (6M/4F, 30 ± 5 yrs) were enrolled in the experimental assessment, which was approved by the Research Ethics Committee for North Jutland (approval N-20190036). The participants were informed about the experiment details, both written and orally, before providing written informed consent.

### 2.6. Experimental Procedure

The experiment comprised repetitive grasp and move tasks with objects of daily living to test the feasibility of the novel approach to prosthesis control and assess whether the participants can use the system efficiently. More specifically, the aim was to evaluate (i) if they could learn how to aim at an indicated target object or object part by manipulating the prosthesis and observing its online response (Figure 1), and (ii) if the system can be successfully used in a difficult scenario such as a cluttered environment. The experiment consisted of two phases: (i) an initial training phase (Phase I), in which the participants practiced how to accurately aim and grasp a sequence of ten individual objects, and (ii) a cluttered environment phase (Phase II), in which the participants grasped four objects arranged in four cluttered scenes. The latter setup was more challenging for computer vision pipeline, in terms of detecting, segmenting and analyzing objects, as well as for the subjects, as they needed to aim to a particular object (or part) within a crowded scene. While in Phase I the subjects practiced strategies to grasp different types of objects, in Phase II, they were expected to apply those strategies in a more challenging context.

**Figure 4:**
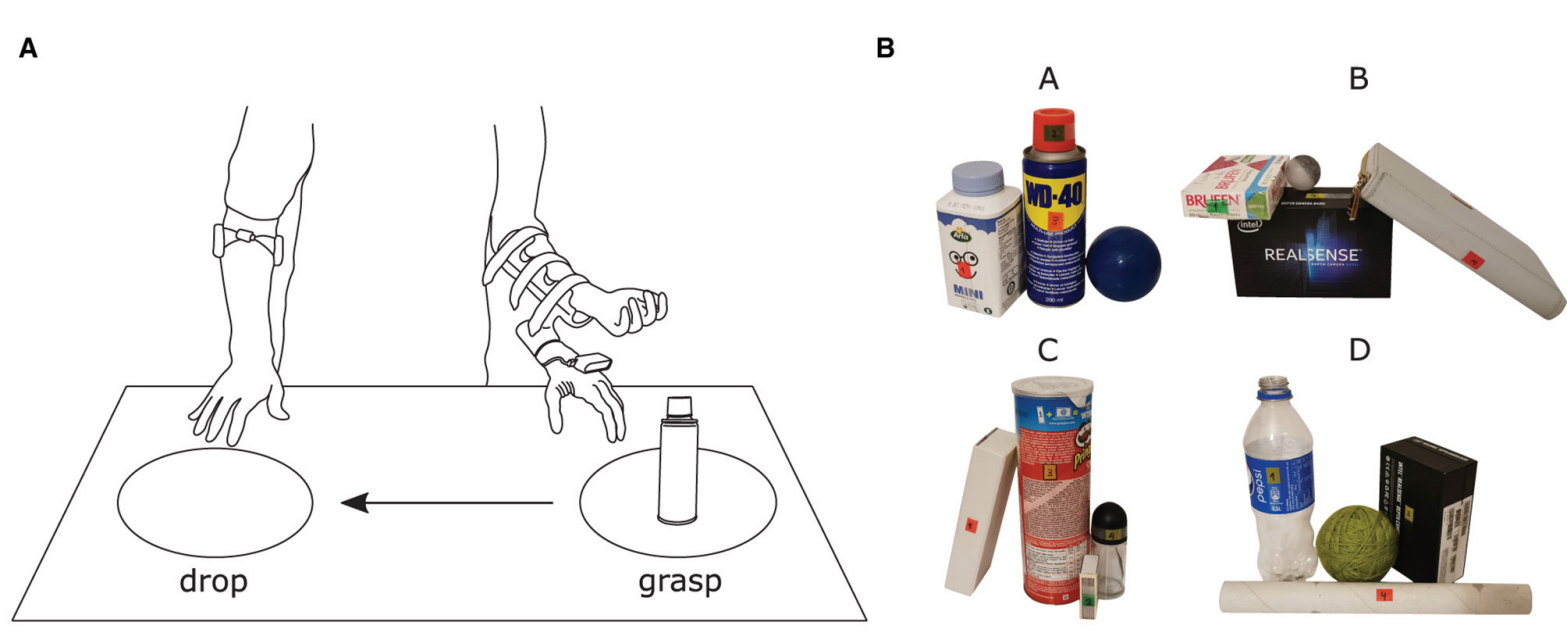
**(A)** Illustration of the experimental environment comprising a table where two marked areas, separated by a distance of 50 cm, defined the positions for grasping and dropping objects. **(B)** All 16 objects organized in four different cluttered scenes (A, B, C, and D). Six new objects were introduced in Phase II and no training was provided for them. The labels placed on each object were used as cues to indicate from where the object should be approached and they also provided the number regarding the grasping order.

Two areas, A and B, separated by a distance of 50 cm were marked on a table (Figure 4A) as the positions for picking up and dropping objects, respectively. While standing in front of the table, the participants were instructed to grasp and move each object placed on the table by an experimenter in the following manner: (1) they moved the prosthesis from the resting place and approached the object placed at position A; (2) adjusted their aim toward the target in order to grasp it according to the defined task requirements; (3) when they judged that the prosthesis was properly configured, they took over the control and closed the hand; and (4) finally, they transported and dropped the object at position B. The participants were instructed to grasp and move the targets as fast as possible and encouraged to try improving their performance over the trials.

The objects included in this study were selected to test the system's robustness and capabilities. Table 1 specifies the dimensions, the orientation of the object with respect to table surface, the side from which it is approached, and the expected grasp type. As can be seen from the table, in Phase I, the task was to grasp objects with distinct sizes and shapes as well as different parts within the same object. Phase II introduced some of the previous objects but in different orientations and in clutters, as well as new objects that the participant had not seen during Phase I.

**Table 1:** List of objects used in the experiment.

**ID**	**Object tag**	**Dim. [mm]**	**P.I angle**	**P.I approach**	**P.II angle**	**P.II approach**	**P.II scene**	**Model**	**Grasp**
01	box_rs_front	52 × **91** × 143	Vertical	Side	Vertical	Side	D3	Cuboid	Palmar
02	cap_wd_spray	**34** × 27	On top of 03	Front	On top of 03	Front	A2	Cylinder	Lateral
03	can_wd_spray	**53** × 132	Vertical	Front	Vertical	Front	A3	Cylinder	Palmar
04	ball_deo_rollon	33	On table	Top	On table	Top	B2	Sphere	Lateral
05	tube_paper	**31** × 300	Vertical	Front	Horizontal	Top	D4	Cylinder	Palmar
06	can_potato_chips	**75** × 230	Horizontal	Top	Vertical	Front	C3	Cylinder	Palmar
07	box_slim_white	**34** × 67 × 175	Vertical	Side	Tilted	Front	C1	Cuboid	Palmar
08	box_rs_top	**52** × 91 × 143	Horizontal	Top	Horizontal	Top	B4	Cuboid	Palmar
09	bottle_pepsi_transp	**63** × 55^*^	Vertical	Front	Vertical	Front	D1	Cylinder	Palmar
10	ball_yarn	85	On table	Top	On table	Top	D2	Sphere	Palmar
11	mini_milk_carton	**55** × 55 × 95	N/A	N/A	Vertical	Side	A1	Cuboid	Palmar
12	ball_blue	65	N/A	N/A	On table	Top	A4	Sphere	Palmar
13	box_brufen_pills	**25** × 65 × 101	N/A	N/A	Horizontal	Front	B1	Cuboid	Lateral
14	large_wallet	**26** × 100 ×200	N/A	N/A	Tilted	Front	B3	Cylinder	Palmar
15	box_matches	**17** × 36 × 58	N/A	N/A	Vertical	Front	C2	Cuboid	Lateral
16	cap_deo_transp	38^*^	N/A	N/A	On table	Top	C4	Sphere	Lateral

The subset 1–10 was used in Phase I (P.I), while all objects were used in Phase II (P.II). Annotation: bold, target grasping dimension; ^*^, object containing a transparent part; N/A, not applicable.

The experimenter stayed in the front of the participant during the experiment, across the table, and indicated the grasp type he/she was supposed to use in the current trial. The target object (or part) and the grasping side were indicated directly on the object by a sticker. The participants were initially familiarized with the system for 10–15 min, and the experimenter provided minimal aiming instructions. The experiment then started with Phase I. The objects were individually placed on the table at position “A” following the order listed in Table 1, which was devised to maximally challenge the system and the participant. More specifically, each time an object was dropped at position “B” (end of trial), the prosthesis would remain in the attained configuration and the following object would always require a change in the wrist angle, even if the grasp type was the same between two consecutive objects. The participants performed five blocks of the ten-objects sequence. After that, the experiment proceeded to Phase II, in which six new objects were added to the object pool (objects 11–16, Table 1). The sixteen objects were then clustered in four scenes of four objects as shown in Figure 4B. Each scene was placed at position A following a randomized order per block. The participants performed three blocks of these four scenes.

### 2.7. Data Analyses

The outcome measure in the experiment was the “time to accomplish the task” (TAT) defined as the time interval from the moment the participant started moving toward the target until he/she released the object. The TAT was measured by the experimenter, who started the timer after giving a verbal command to the subject to begin the task and stopped the timer when he heard a beep generated by the software to indicate the loss of contact force. To assess whether the participants improved across blocks, the median TAT was computed for each subject across all trials/objects in each block (assessment 1). To test if the subjects improved the performance when grasping a particular object across blocks, the median TAT for each object over all participants was computed in each block (assessment 2). Finally, to determine whether grasping some objects was more challenging overall, the median TAT was computed for each object over all participants and blocks (assessment 3).

A Shapiro-Wilk normality test was used to assess if the data was normally distributed. After finding that the data were not normally distributed, a Friedman test was used to evaluate if there was statistically significant difference in performances overall, for the multiple conditions in each assessment. If the test indicated significant difference, *post-hoc* pairwise comparisons were performed using either the Wilcoxon signed rank test with Bonferroni correction (assessments 1 and 2), or the Tukey's Honestly Significant Difference test (assessment 3).

Lastly, the differences in performance when handling the ten objects that were present in both Phase I and II were also investigated. The median TAT for each object obtained in Phase I was statistically compared to that achieved in Phase II using the Wilcoxon signed rank test. This evaluated whether aiming at and grasping a target object or object part in a cluttered environment posed an extra level of difficulty. The threshold for statistical significance was set at *p* ≤ 0.05 and the results are reported in the text as M{IQR}, where M is the median and IQR the interquartile range.

## 3. Results

### 3.1. System Performance and Versatility

The computer vision processing pipeline allowed the system to function at frame rates between 5 and 16 Hz. The frame rate was variable as the processing time depended on several factors (e.g., object size, shape, and clutter, as well as aiming) and could therefore vary across different objects and trials. For instance, large cuboids were the most computationally heavy (larger point clouds), resulting in processing times up to 200 ms (5 Hz), whereas smaller cylinder and spherical objects (smaller point clouds) could be processed, on average, in 60 ms (16 Hz). Moreover, all objects but one were almost always successfully fitted to their corresponding shape model (overall accuracy of ~97%). The smallest cylinder object, 02 (spray cap), was mistaken for a spherical object 20% of the times due to the reduced amount of points captured, meaning that 20% of the times the sphere model presented a better fitting percentage than the cylinder model. Yet, the implemented majority voting approach enabled the system to function as expected in terms of grasp size. Finally, it should be noted that the size of the smallest spherical and cylindrical objects (02—spray cap, 04—small ball, and 16—spherical cap) tended to be overestimated by ~1 cm on average, which resulted in ~20% of grasp type misclassifications especially for object 16 due to one of the dimension rules (*d* ≤ 5) imposed by the grasp selection decision tree.

As soon as the automatic controller generated a new decision (desired hand configuration), it sent the commands to the hand for the grasp type, size, and wrist rotation. The movement velocity of the robotic hand was, however, limited to at most a half of its maximum speed (i.e., 12.5 rpm for wrist rotation and 162.5 mm/s for gripping speed) to avoid an uncanny feeling to the user and facilitate the interpretation of the system behavior. The pilot tests demonstrated that such speeds also successfully accommodated the computer vision pipeline processing rates.

**Figure 5:**
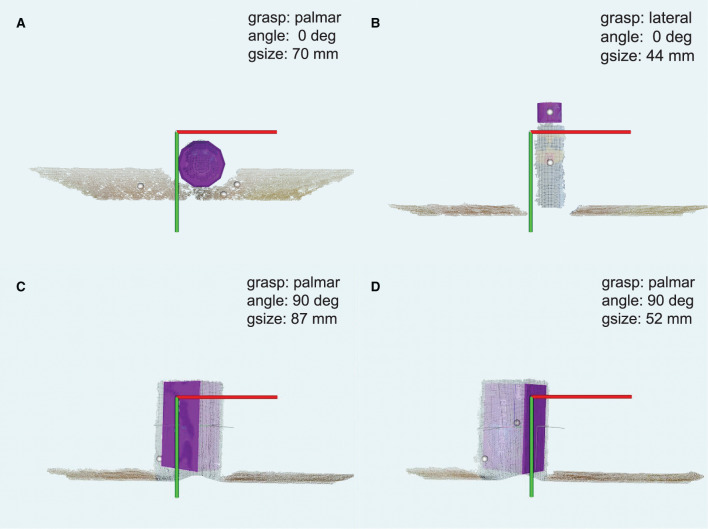
Snapshots captured while grasping example objects in Phase I. The system correctly detected spherical, cylindrical and cube shaped objects or object parts. The point cloud centroids are rendered as small white spheres in the figures. The point cloud with the closest centroid to the Z'-axis of the hand/camera frame was selected for fitting (purple shape primitives). The user selected the ball **(A)** and plastic cap **(B)** by aiming at the vicinity of these objects. In **(C,D)**, the user marked different faces of the same cuboid object (highlighted shades). Each panel reports the selected grasp type, size, and wrist orientation. The coordinate frame represents the X'OY' plane of the camera.

**Figure 6:**
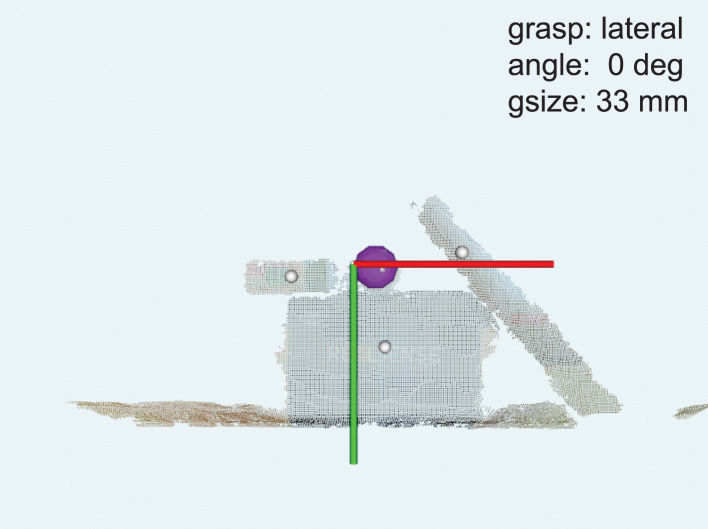
Grasping an object from a cluttered scene in Phase II. The user successfully marked a small ball for grasping. The annotations are the same as in Figure 5.

**Figure 7:**
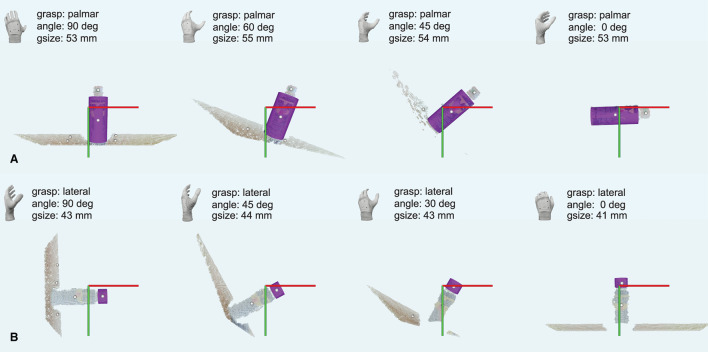
A sequence of snapshots taken while the system approached two different cylindrical parts of the same object: the body **(A)** and the cap **(B)** of a spray can. The annotations are the same as in Figure 5. Note that the can body was approached with a fully pronated prosthetic hand, while the cap was approached with the hand in the neutral position.

Figures 5–7 depict illustrative examples of the system operation recorded during the experiment. The snapshots in Figure 5 are captured during Phase I and they report captured point clouds and resulting system decisions, namely, estimated grasp type, size, and wrist rotation, when grasping three different objects from Table 1 (10, 02, and 01). Figures 5A,B show that the system can detect a target object (ball, 10) and a part of the object (plastic cap, 02) while it is not necessary for the subject to aim directly at the target but only in its vicinity. Figures 5C,D demonstrate that the subject can use the system to grasp different sides of a cuboid object (box, 01) by adjusting the aiming, where depending on the dimensions of the two sides this can lead to different grasp sizes and/or types. A complete run over a block of ten objects in Phase I was recorded and provided as Supplementary Video 1. In Figure 6, a cluttered scene from Phase II is shown, in which the subject successfully marked a single small object, tightly surrounded by much larger objects. This is further illustrated in Supplementary Video 2, which shows the use of the system to grasp objects arranged in the cluttered scenes of Phase II. The adjustment of wrist orientation is depicted in Figure 7. In Figure 7A, the participant aimed at the body of the spray can (object 03), while in Figure 7B, the target was the plastic cap (object 02). The first snapshots show that the two targets were approached with the hand (camera) in different initial positions, hence different orientation of the camera with respect to the object. Snapshots 2–4 show the rotation of the prosthesis from initial into final position, namely, hand horizontal. Note that the computer vision loop operated continuously, which means that the frames were continuously captured and reanalyzed while the hand was moving. If the participant, at any moment, changed the target, the hand would react momentarily. This is shown in Supplementary Video 3, where a subject uses the system to consecutively target at several objects placed on a table or stacked vertically. Each time the hand is pointed toward a different object, the prosthesis automatically readjusts its configuration. Finally, Supplementary Video 4 shows that the system can grasp objects that are not necessarily placed on a table surface. In the video, a subject uses the prosthesis to grasp objects that are handed to him by another person.

### 3.2. Phase I: Training

**Figure 8:**
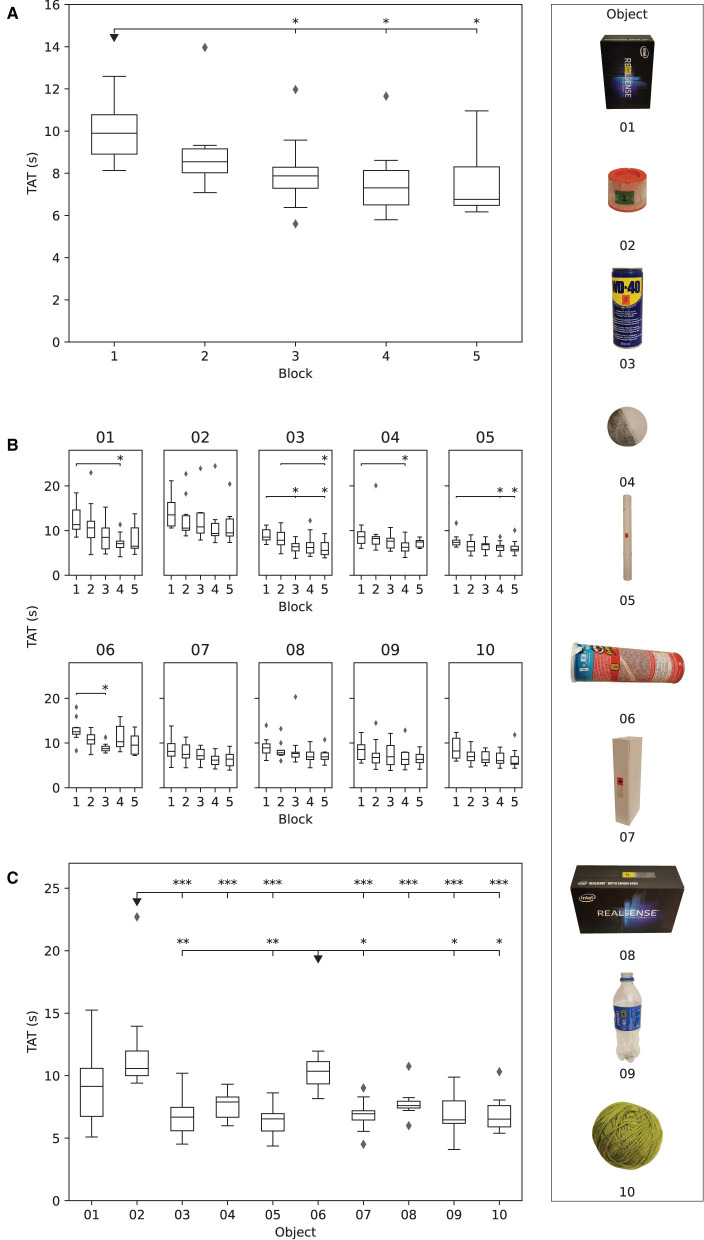
The results obtained in Phase I. The boxplots show: **(A)** median time to accomplish the task (TAT) for each subject in each block, **(B)** median TAT of all subjects for each object in each block, and **(C)** median TAT per object across all blocks. The stars indicate statistical significance (*p < 0.5; **p < 0.01; ***p < 0.001).

A general trend of decreasing TAT was observed across blocks in Phase I as shown in Figure 8A. The TAT in the third (7.9{1.0}s), fourth (7.3{1.6}s), and fifth (6.8{1.8}s) block was significantly lower (*p* ≤ 0.05) compared to that in the first block (9.9{1.9}s). The reduction in the median TAT between the first and the fifth block was ~32%.

The median TAT per individual object across blocks is shown in Figure 8B. There was a decreasing trend in TAT across blocks for all objects, but the improvement in performance was statistically significant (*p* ≤ 0.05) for objects 01 (larger box), 03 (spray can), 04 (small ball), 05 (paper tube), and 06 (potato chips can). The median TAT for objects 01 (larger box, 11.3{4.3}s), 02 (spray cap, 13.5{5.26}s), and 06 (potato chips can, 12.5{1.4}s) was initially above 10 s, i.e., in the first block, indicating that those objects were more difficult to grasp. Nevertheless, object 01 was also characterized with the greatest decrease (11.4{4.3}–6.5{4.6}s) in median TAT over blocks. The object with the least improvement in performance from the first (8.6{2.6}s) to the fifth block (7.4{1.4}s) was 04 (small ball), followed by objects 05 (tube paper) and 07 (slim box), in which TAT decreased from 7.4{1.1} to 5.8{1.1}s and from 8.1{2.8} to 6.4{2.5}s, respectively.

Figure 8C depicts the comparison of the overall median TAT for each object in Phase I. The participants were significantly slower when grasping objects 02 (spray cap, *p* ≤ 0.001) and 06 (potato chips can, *p* ≤ 0.05 or *p* ≤ 0.01) compared to most of the remaining objects. These two objects were therefore most difficult for the participants to handle.

### 3.3. Phase II: Cluttered Scenes

**Figure 9:**
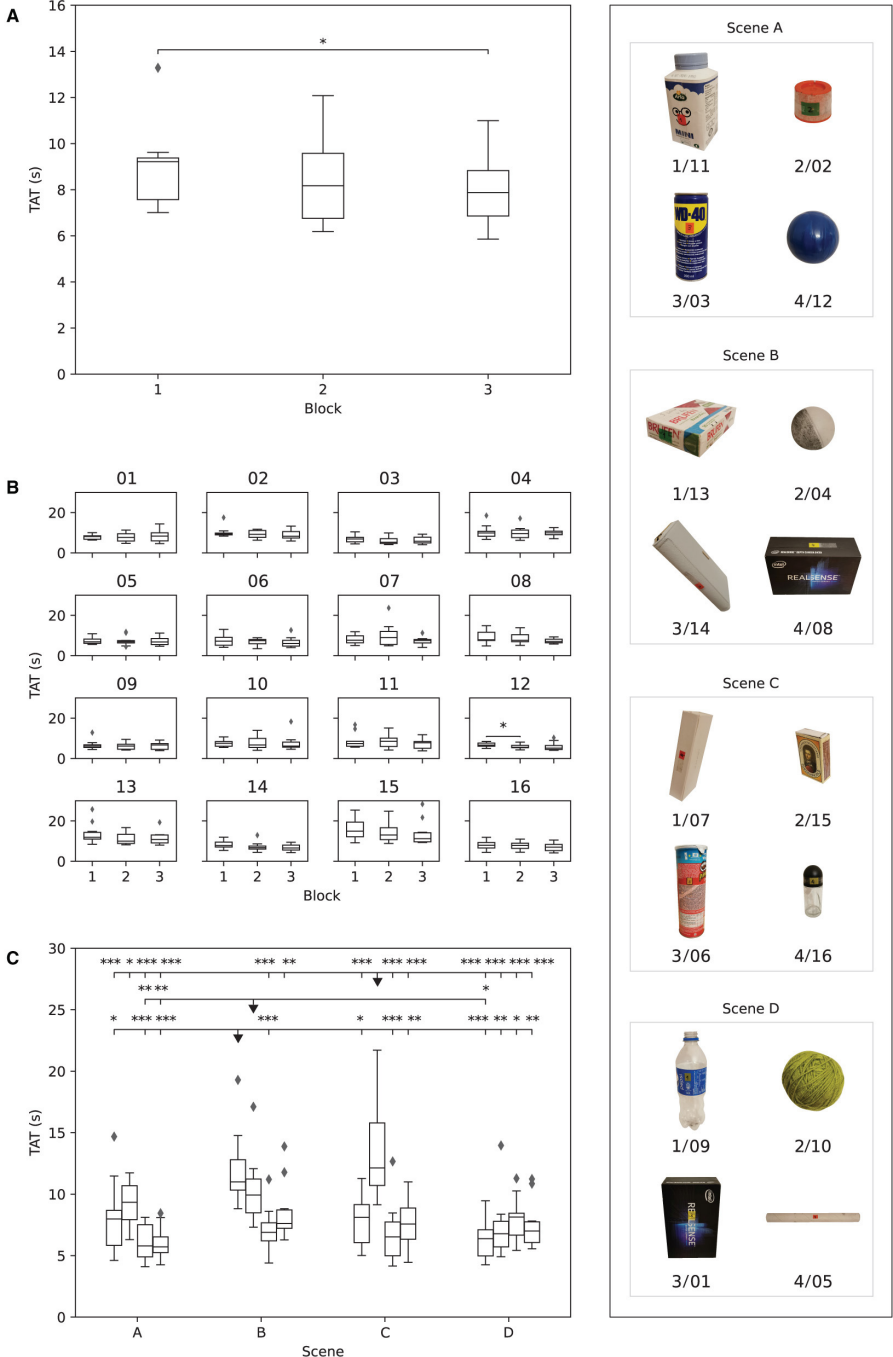
The results obtained in Phase II. The boxplots show: **(A)** median time to accomplish the task (TAT) for each subject in each block, **(B)** median TAT of all subjects for each object in each block, and **(C)** median TAT per object across all blocks. The boxplots (objects) in each scene are ordered as in Table 1. The stars indicate statistical significance (*p < 0.5; **p < 0.01; ***p < 0.001).

The general trend of decreasing TAT was also present across the three blocks of Phase II, as reported in Figure 9A. The statistically significant decrease (*p* ≤ 0.05) in median TAT was observed between the first (9.2{1.8} s) and the last (7.8{1.9} s) block.

At the object level (Figure 9B), the only statistically significant improvement in performance across blocks (*p* ≤ 0.05) was registered for object 12 (blue ball), with a median TAT decrease from the first (6.6{1.5} s) to the second (5.5{1.2} s) block.

The differences in median TAT between the objects in Phase II are reported in Figure 9C. Three objects (13/B1—box of pills, 04/B2—small ball, and 15/C2—match box) were markedly different than the others, as their TAT was statistically significantly higher compared to most other objects. These three objects were therefore the most challenging to grasp while in clutters, especially object 15/C2 (match box). Two of these objects, namely 13/B1 and the 15/C2, were not presented and therefore not trained by the participants during Phase I.

### 3.4. Between Phases Comparison

**Figure 10:**
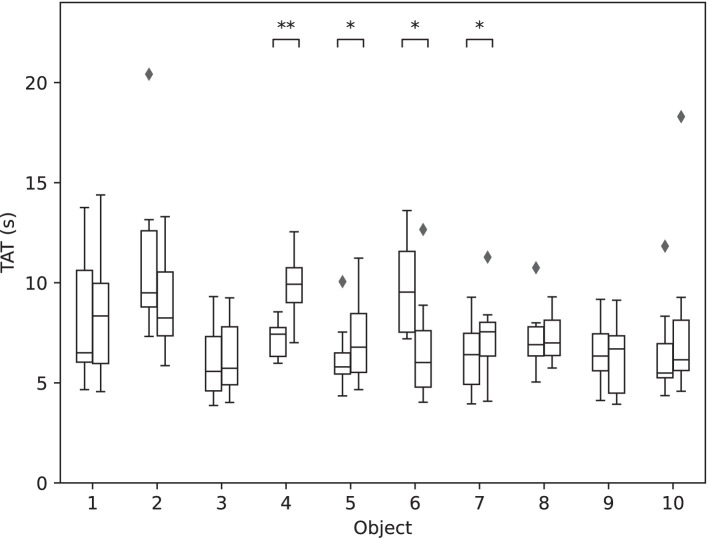
The performance for each of the 10 objects in Phase I vs. Phase II. The boxplots show median TAT per object across all blocks in Phase I (left-hand side) and Phase II (right-hand side). The stars indicate statistical significance (*p < 0.5; **p < 0.01).

As shown in Figure 10, four of the objects handled in both Phase I and Phase II showed statistically significant differences in TAT performance: 04 (small ball), 05 (paper tube), 06 (potato chips can), and 07 (slim box). With the exception of object 06 (potato chips can) that showed a significant decrease in TAT, the other three exhibited a significant increase in TAT, showing that cluttered environments in some cases worsened the performance, despite the previous training.

## 4. Discussion

The present work investigated the feasibility of a novel depth-embedded, yet robust and reliable, approach for semi-autonomous prosthesis control using computer vision. The tests conducted in 10 participants aimed to assess (1) the capabilities of the system enabled by the developed depth processing and decision-making pipeline, and (2) the interaction of the subject with the semi-autonomous system that constantly reacts to his/her movements and the environment, i.e., the user-prosthesis integration.

The experiments demonstrated that the novel context-aware system was indeed effective. Despite the camera was placed on the hand, thus limiting the field-of-view, the system guided by the subject (aiming) successfully automated the prosthesis pre-shaping phase while grasping a diverse set of targets (Table 1). The set included objects and object parts of various shapes and sizes, individually, and in clutters. The participants successfully handled all target objects, and the system reacted appropriately when the objects were approached from different sides (top or front) and orientations. They could use the system to grasp specific object parts, when the target was isolated on the table or placed within a cluttered scene.

The experimental results demonstrated a successful user-prosthesis interaction. The participants learned how to aim with the camera from distinct perspectives/positions, regardless of the prosthesis wrist rotation (prosthetic hand horizontal or vertical) as shown in Figure 7. They also learned how to “read” the prosthesis behavior while it reacted to how they aimed at a given target object or object part.

The participants improved consistently during the grasp and move tasks in Phase I (section 3.2), and they became faster the more they practiced. As they learned the system behavior, most of them started anticipating prosthesis movements during aiming and they took over control earlier in the task. They did not wait for the prosthesis to converge to a perfect alignment for grasping a particular target. Once they perceived that the prosthesis was in an approximately suitable position for grasping, they took control and grasped the object.

Nevertheless, some target objects or object parts were initially more difficult to handle than others (Figure 8B). For instance, object 01 (large box) was the largest in the set, and it was more challenging to grasp stably after aiming because of the finger configuration of Michelangelo hand. The presence of an extra wrist flexion/extension DoF may improve this. Object 04 (small ball) was the smallest object and thus difficult to properly aim at, often leading to a point cloud of lower quality, which was sometimes mixed up with the larger point cloud of the table surface in the background. Object 06 (potato chips can) is a long cylindrical object lying on the table for which the participants had to perform a slight elbow compensation, as mentioned earlier in section 2.4. Still, the participants significantly improved in all these cases as they learned how to handle them. However, the same did not occur when grasping object 02 (spray cap). This case posed a particular challenge during the training given that it included a small object (similar to object 04), which was additionally a part of another object (03, spray can). Hence, successful grasping required precise aiming, as a small deviation would cause the system to recognize the body of the spray can as the target. As explained in section 2.3, the system is robust enough to select a target even if the camera was not directly aimed at it. This simplified the aiming process for the participants; however, in the case of object 02, such assistance was still not enough to lead to a significantly better performance with training.

The participants improved performance over blocks in Phase II (Figure 9A). They acquired strategies on how to use the system to grasp isolated objects in Phase I, and they employed those strategies successfully in Phase II to grasp the same objects when they were placed within a cluttered scene. Nevertheless, aiming in Phase II was more challenging as the objects in a cluttered scene were close to each other, and a small deviation in aiming might cause the system to pick a centroid that belongs to another object close to the desired target. This was especially expressed for the smallest objects (Figure 9C), such as 13/B1 (box of pills), 04/B2 (small ball) 15/C2, matches box).

When comparing the same objects across the two phases (Figure 10), the largest difference was obtained for object 04 (small ball) and object 06 (potato chips can). For object 04, the performance substantially decreased when it was placed in the cluttered scene due to its small size. Contrarily, object 06 was handled faster when in a clutter; this “paradoxical” result can be explained by the fact that in Phase II, the object was placed vertically—a long vertical object was an easy target for aiming.

The first system in the literature that used a camera placed on the hand relied on a laser to mark the target object (Došen et al., 2010). This was an effective approach, but might be challenging to translate into clinical application (e.g., user acceptance). In another study (Ghazaei et al., 2017), the subjects were first trained how to aim using visual feedback on the computer screen and later on, they were able to use the system when the feedback was removed. Compared to these systems, the solution presented here used depth sensing instead of an RGB camera, allowing thereby more flexibility in reacting to the environment (e.g., identifying object parts, composite objects). In addition, in both RGB-based systems as well as in previous solutions that used depth sensing (Markovic et al., 2014, 2015; Mouchoux et al., 2021), the user needed to activate the automatic controller explicitly (myoelectric command), which then processed the scene and generated a “one-shot” response. In this novel system, however, the automatic control is active from start, adjusting the prosthesis configuration continuously using visual servoing based on user aiming.

The present study showed that prosthesis motion in response to subject movements provided meaningful feedback about the aiming process. The participants judged whether the prosthesis achieved an appropriate configuration for grasping by estimating grasp type and wrist rotation. If this was appropriate, they would proceed and grasp, and if not, they would try to reposition the prosthesis to “trigger” the right target. The participants reported they were not always able to interpret the prosthesis movement correctly (e.g., distinguish the momentary grasp type), especially in the initial trials. A simple approach to overcome this drawback, could be to provide a cue about the grasping type and wrist movement through electrotactile or vibrotactile feedback (Stephens-Fripp et al., 2018; Sensinger and Došen, 2020). This could also enable the subject to decrease the visual attention to the prosthesis, which is presently required to monitor the aiming.

The camera is placed on the dorsal aspect of the prosthetic hand with an offset with respect to the axis of the wrist joint. Therefore, when the hand rotates, the participants needed to slightly adjust their aim to maintain the selected target object in the focus of the camera. A self-contained system designed from scratch would allow to overcome this problem since the components could be positioned in the most convenient manner. For instance, the camera could be integrated into the hand so that its optical axis is aligned to the axis of the wrist joint. Another interesting point that could be considered is the integration of more cameras (depth and/or RGB) to maximize the assessment of the scene as well as simplify the aiming. Currently, the system only handles depth data, which is approximated by one of three primitive shapes (sphere, cylinder, or cuboid). Nevertheless, as the system can isolate whole objects but also object parts, this means that it can still handle a diverse set of objects with simple but also composite shapes (i.e., theoretically, any combination of the three primitives). Even when the object does not resemble one of these three shapes, the system is versatile enough to pick the best approximation among the three models.

Overall, this study demonstrated that a continuous semi-autonomous control can be implemented using a sensor placement on the prosthetic hand. Hence, the user does not need to wear an extra piece of equipment, as for instance in similar systems presented recently (Markovic et al., 2014, 2015; Mouchoux et al., 2021). This solution has similar capabilities to those advanced implementations, such as the ability to handle cluttered scenes, and such functionality is achieved using a compact setup. The pervasive analysis of the environment from the user perspective, as proposed in those studies, eliminates the need for aiming. In the present study, the participant has to engage during aiming, but this also gives him/her a unique possibility to “select” not only the whole object (as in all other cases in literature) but also an object part (e.g., a cap on the top). To grasp an object, the subjects need to orient the prosthesis properly, but the semi-autonomous system commands the prosthesis, which decreases the cognitive burden of controlling multiple DoF as well as the muscle effort, i.e., the amount of muscle activation. Importantly, the conducted tests demonstrated that the subjects successfully learned to aim with the system after a brief training. Some objects were more or less challenging, hence requiring more or less time to handle, but none of the objects was too difficult for the subjects to prevent the eventual successful completion of the task.

The proposed approach has not been, however, compared to the conventional interfaces (e.g., direct control and pattern classification) since the advantages of semi-autonomous control have been demonstrated before (Markovic et al., 2015; Mouchoux et al., 2021). Thus, this system should not be regarded as an ultimate solution but rather as an alternative to conventional control. If such control framework is implemented in a prosthesis with an embedded “on-hand” sensor, a self-contained system can potentially be built. To achieve this, the proposed framework would need to be packaged and deployed onto an embedded platform, such as the Nvidia's Jetson, as used in a recent work (Ragusa et al., 2021). While such boards are still more powerful than standard clinically used prosthesis controllers, this is likely to change in the near future as new designs with embedded cameras are being presented and tested (Choudhry and Khan, 2018; Weiner et al., 2018). The present system was not tested on amputees, however, from the functional viewpoint, the use of the system by an amputee is not different compared to an able-bodied subject. In fact, since the socket is aligned with the arm (residual limb), the aiming could be even easier. For manual control, the system relies on the classic two-channel EMG interface, which is a clinical standard and hence known to prosthesis users. Therefore, we assume that an amputee could use the system after a brief training, similarly to able-bodied subjects tested in this study. Nevertheless, subjective experience and clinical utility of this approach still needs to be investigated in a population of prosthesis users.

## 5. Conclusion

This work presents a depth-embedded system comprising a prosthesis with a dorsally mounted depth camera, which enables semi-autonomous control of grasping actions. The experimental assessment showed that the participants could use the system to grasp a very diverse set of objects or object parts, approached from different directions and placed individually or in cluttered environments. The participants easily learned to adapt to the system and “read” its online behavior (response to targeted objects), and they significantly improved their performance after a brief training.

## Data Availability Statement

The raw data supporting the conclusions of this article will be made available by the authors, without undue reservation.

## Ethics Statement

The studies involving human participants were reviewed and approved by Research Ethics Committee for North Jutland. The patients/participants provided their written informed consent to participate in this study.

## Author Contributions

SD conceptualized the study. MC implemented the computational framework. Both authors contributed to designing the experimental setup, to analysing the data, and to the writing of the manuscript. Both authors contributed to the article and approved the submitted version.

## Funding

The present study was supported by the project ROBIN: Robust bidirectional human-machine interface for natural control and feedback in hand prostheses (8022-00243A) funded by the Independent Research Fund Denmark.

## Conflict of Interest

The authors declare that the research was conducted in the absence of any commercial or financial relationships that could be construed as a potential conflict of interest.

## Publisher's Note

All claims expressed in this article are solely those of the authors and do not necessarily represent those of their affiliated organizations, or those of the publisher, the editors and the reviewers. Any product that may be evaluated in this article, or claim that may be made by its manufacturer, is not guaranteed or endorsed by the publisher.
